# Structural Aspects LiNbO_3_ Nanoparticles and Their Ferromagnetic Properties

**DOI:** 10.3390/ma7117217

**Published:** 2014-10-28

**Authors:** Carlos A. Diaz-Moreno, Rurik Farias-Mancilla, Jose T. Elizalde-Galindo, Jesus González-Hernández, Abel Hurtado-Macias, Daniel Bahena, Miguel José-Yacamán, Manuel Ramos

**Affiliations:** 1 Departamento de Física y Matemáticas, Instituto de Ingeniería y Tecnología, Universidad Autónoma de Cd. Juárez, Avenida del Charro #450 N. Cd. Juárez, Chihuahua, C.P. 32310, Mexico; E-Mails: rurik.farias@uacj.mx (R.F.-M.); jose.elizalde@uacj.mx (J.T.E.-G.); 2 Materials Research and Technology Institute, University of Texas at El Paso, 500 W, University Ave, El Paso, TX 79968, USA; E-Mail: carlos.alejandro.diaz.moreno@gmail.com; 3 Kleberg Advanced Microscopy Center, University of Texas at San Antonio, One UTSA Circle, San Antonio, TX 78249, USA; E-Mails: d.bahena.u@gmail.com (D.B); miguel.yacaman@utsa.edu (M.J.-Y.); 4 Centro de Investigación en Materiales Avanzados S.C., Laboratorio Nacional de Nanotecnología, Miguel de Cervantes 120, Complejo Industrial Chihuahua, Chihuahua, Apdo. Postal 31109, Mexico; E-Mails: jesus.gonzalez@cimav.edu.mx (J.G.-H.); abel.hurtado@cimav.edu.mx (A.H.-M.)

**Keywords:** scanning transmission electron microscopy (STEM), nanoparticles, lithium niobate, ferroelectric, ferromagnetic

## Abstract

We present a solid-state synthesis of ferromagnetic lithium niobate nanoparticles (LiNbO_3_) and their corresponding structural aspects. In order to investigate the effect of heat treatments, two batches of samples with a heat-treated (HT) and non-heat-treated (nHT) reduction at 650 °C in 5% of hydrogen/argon were considered to investigate the multiferroic properties and their corresponding structural aspects; using magnetometry and scanning transmission electron microscopy (STEM). Results indicate the existence of ferromagnetic domains with a magnetic moment per unit cell of 5.24 × 10^−3^ μB; caused mainly due to voids and defects on the nanoparticle surface, as confirmed by STEM measurements.

## 1. Introduction

Currently, it is relatively easy to find information regarding perovskite structures, such as ABO_3_ compounds (*i.e.*, PbTiO_3_ (PT), BaTiO_3_ (BT), Pb(Zr,Ti)O_3_ (PZT) and LiNbO_3_ (LNO)) as principal materials for nonlinear optical applications [1]. The study of those particular systems has arisen due to their large dielectric constant values [2]. In the past four years, crystalline lithium niobate (LNO) has been considered a material with enormous potential for materials engineering applications, such as optoelectronics material [3]. It is relatively easy to find extensive information in the literature about routes for synthesis and characterization [4], along with experimental techniques to measure the ferromagnetic/ferroelectric response, especially nano-scaled structures (spheres, nanorods) [5,6], as well as their corresponding electronic structure using computational methods [7]. In the search for a fundamental understanding of ferromagnetism in the LNO crystalline structure, theoretical hypotheses have been constituted according to Wei *et al.*, who attributed it to an addition of contaminants into the LNO structure (the effect of dopants). This seems to agitate the Li-Nb-O electronic structure [8]. Kong *et al.* argue the formation of ferromagnetism when implanting Ni^+^, Fe^+^ or Mn^+^ and Co^+^ ions into the LiNbO_3_ crystal structure [9]. A preliminary conclusion made by Sundaseran and Rao indicates that ferromagnetic properties can be caused in LNO nanostructures by the formation of oxygen vacancies near the surface [10], also because of polarization values of 70 μC/cm^2^ found in LNO crystals at room temperature, with the existence of the ferroelectric phase at high transition temperature values of 1483 K [11]. The latter, an important path to design and determine the fundamental study of the magnetic behavior, due to structural defects, has led to the search for new materials. Such is the case of the high temperature ferromagnetic behavior reported in LiNbO_3_ nanocrystal, due to heat treatment reduction, causing defect structures near the surface of nanoparticles [12]. One can find in the literature two main manuscripts regarding multiferroic properties in LiNbO_3_: Song *et al.*, who reported multiferroic properties when LiNbO_3_ was doped with cobalt (Co), calling it a two-phase material [11]. Additionally, the other work done by this group reported multiferroic properties in one phase in nanoparticles of LiNbO_3_ [13]. Furthermore, the recent advances in small-scaled electronics combined with computational equipment allowed microscopy analysis with point resolutions of 0.08~0.11 nm and a magnification of 150,000,000× in STEM Cs-corrected instruments [14]; consequently, new information regarding nanoparticle structural aspects is possible to obtain using these characterization techniques. For example, using microanalysis principles, Borisevich *et al.* were able to detect with high success the localization sites for oxygen vacancies on metallic interfaces [15]. Furthermore, the usage of irradiation energy from a field emission gun source while surveying specimens in STEM microanalysis allowed the formation of new crystallographic phases, as presented by Sepulveda-Guzman *et al.*, in the formation of bismuth nanoparticles at 200 kV using a NaBiO_3_ powder precursor [16]. Here, we present the solid-state reaction synthesis of lithium niobate nanoparticles from lithium carbonate and niobium pentoxide precursors, followed by heat treatments at 650 °C in a hydrogen atmosphere. In order to investigate both the ferromagnetic properties and structural aspects, two batches of samples (heat treated and non-heat treated) were considered for powder X-ray diffraction, Cs-corrected STEM and magnetometry analysis.

## 2. Results and Discussion

### 2.1. Differential Scanning Calorimetry

**Figure 1:**
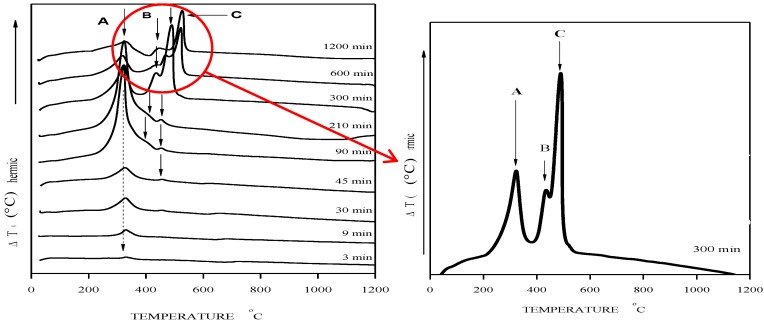
Differential scanning calorimetry of Lithium Niobate (LiNbO_3_) for 3 to 1200 min of milling. Inset: exothermic peaks for 300 min of milling time.

In order to find the temperature window at which the formation of lithium niobate exists during calcination process, a series of differential scanning calorimetry (DSC) was done for the solid-state reaction with various milling times ranging from 2 to 1200 min. The results indicate the existence of exothermic reactions as marked at the A, B and C sites, as presented in Figure 1. The main change observed corresponds to 300 min at 317 °C, 435 °C and 486 °C, as presented in the inset of Figure 1 (three main peaks).

One brief explanation could be that a larger milling time (300 min) can provoke the increase of the surface energy and, therefore, the lower energy or temperature required for crystallization, contrary to the low milling time, as observed, with no peaks near 300 °C to 600 °C.

### 2.2. Powder X-ray Diffraction Pattern

**Figure 2:**
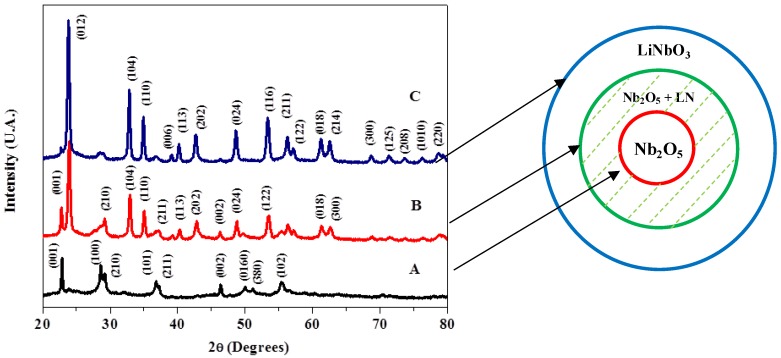
Powder XRD for samples prepared at 300 min of milling and corresponding to calcination temperatures at (**A**) 350 °C, (**B**) 450 °C and (**C**) 650 °C. Inset: three phases generated for different calcination temperatures, which determine the ideal temperature conditions to form spherical LiNbO_3_ nanoparticles, as confirmed by XRD measurements.

To correlate data found from DSC measurements, a series of powder X-ray diffraction was performed, focusing on samples processed at 300 min of milling and calcination at 350 °C, 450 °C and 650 °C. The results indicate a seed material corresponding to niobium pentoxide (Nb_2_O_5_) as the observed poor crystallinity presented in Figure 2A; then, a mix phase found, made out of Nb_2_O_5_ and lithium niobate (LiNbO_3_), as presented in Figure 2B; finally, a pure crystalline phase of LiNbO_3_ is found with (012), (104) and (110) principal refractions in Figure 2C, as indexed using ICDD #00-074-2238 cards, in agreement with Shimada *et al.* [17].

### 2.3. Magnetometry Measurements

**Figure 3:**
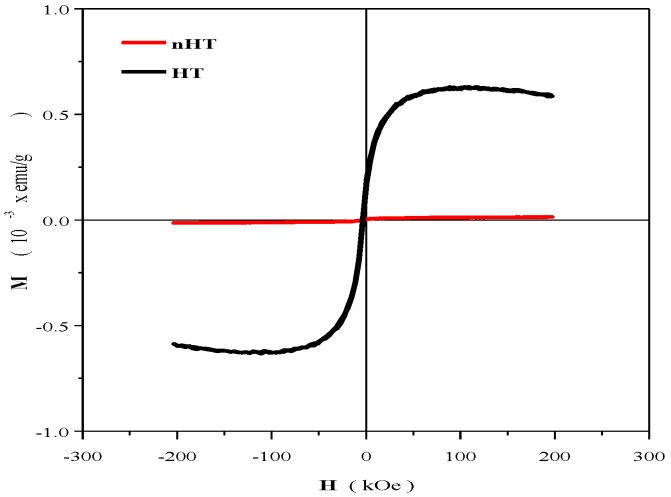
Magnetization curves measured at 9T and 300 K for the sample under heat treatment reduction at 650 °C and non-heat treatment conditions.

After the calcination process, just the sample under calcination at 650 °C was subjected to an additional heat treatment reduction at 650 °C in hydrogen/argon (5%/95%) for 20 min. A grey coloration is observed in comparison with the white powder before the sintering process. The two samples were subjected to magnetization measurements at 9T external field at room temperature (300 K) using the Quantum Design physical properties measurement equipment. A hysteresis loop is observed for the sample under heat treatment reduction, as presented in Figure 3; that magnetization is attributed practically to a non-coercivity caused due to structural defects or voids in the LiNbO_3_ structure. Coey *et al.* explained that these phenomena could be attributed to magnetic regions rich in defects, which tend to concentrate at interfaces and grain boundaries [18]; which, in the case of the sample without heat treatment reduction, do not present a hysteresis loop; consequently, a non-magnetic behavior.

### 2.4. Structural Aspects by Cs-Corrected Scanning Electron Microscope

**Figure 4:**
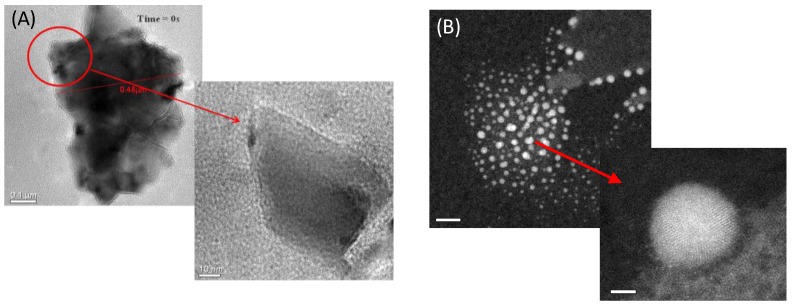
(**A**) Dark field and bright field STEM images corresponding to LiNbO_3_ without heat-treatment (scale bar 0.1 μm) and (**B**) with heat treatment reduction at 650 °C (scale bar 20 nm). (**A**) Mono-dispersed Lithium Niobate and (**B**) nanoparticle of Lithium Niobate.

A small amount of samples that was subjected to magnetization measurements (heat-treated (HT) and non-heat-treated (nHT)) was dispersed in ethanol, and one drop of the solution was deposited onto a silicon-carbide 200 mesh TEM grid and dried at room temperature. For the sample without heat treatment, mono-dispersed sheets of LiNbO_3_ and a well-formed spherical shape for samples with heat treatment were observed, as presented in Figure 4.

**Figure 5:**
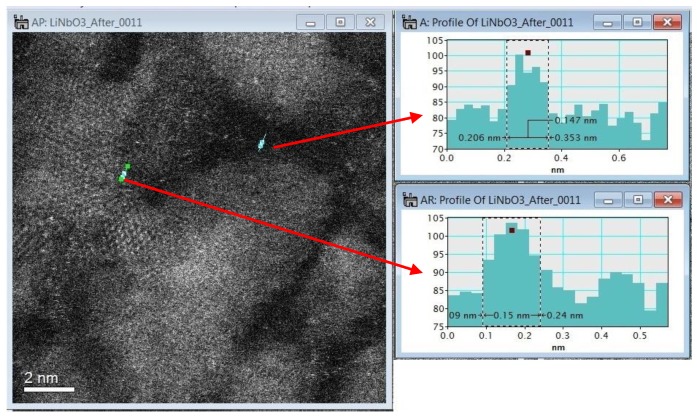
Dark field and bright field STEM image for LiNbO_3_ with the heat treatment reduction at 650 °C; the measurements displayed correspond to single atoms of lithium and niobium, 0.155 nm and 0.147 nm, respectively, as measured using the Gatan^®^ DigitalMicrograph software.

The formation of spherical shaped nanoparticles is mainly attributed to the heat treatment reduction, which also causes some structural defects for those samples, as described previously by meaning of the quantification of those vacancies using field emission gun electron microscopy, along with density functional theory calculations, as reported in [12]. Nanoparticles were unstable under observation, which, in most cases, caused the diffusion of atoms near the surface, as presented in Figure 5, for samples that received previous heat treatments at 650 °C.

**Figure 6:**
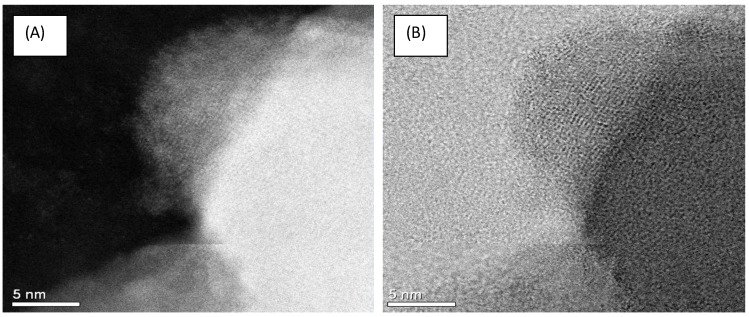
(**A**) Dark field and (**B**) Bright field STEM image of LiNbO_3_ without the heat treatment reduction at 650 °C; one can observe the formation of spherical shaped nanoparticles; one can also observe the formation of the spherical shape as being caused by the e-beam after 10 min of observation at 200 kV.

On the contrary, the formation of spherical shaped nanoparticles was observed after 20 min of observation in STEM, for the sample without heat treatment. Figure 6 presents those observations; one can also observe a non-diffusion of lithium and niobium atoms near the surface, as previously presented in Figure 5. Sepulveda-Guzman *et al.* presented a similar microanalysis study, for the sintering of bismuth nanoparticles, attributed to the segregation of atoms, due to electron beam irradiation when performing *in situ* High Resolution Transmission Electron Microscope (HRTEM) experiments [16].

## 3. Experimental Section

### 3.1. LiNbO_3_ Nanoparticle Preparation

Using 0.6525 g of lithium carbonate (Li_2_CO_3_) and 2.3474 g of niobium oxide (Nb_2_O_5_) with high purity (99.99%), commercially available from Alfa Aesar, as precursors, a mixture is produced by mechanical milling in a SPEX Series 8000 M with a mixture/ball relation of 0.1. The milling time used was 300 min, followed by a heat treatment at 650 °C in a Thermolite 2136 in air atmosphere to obtain the Lithium Niobate (LiNbO_3_) nanoparticles. Stoichiometric (equimolar quantity) LNO powders were prepared by using high-energy milling (SPEX 800 mixer, Metuchen, NJ, USA) with a nylon vial and zirconia ceramic balls. The reaction follows the stoichiometric chemical formula below:

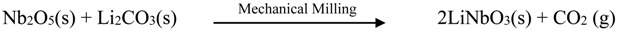
(1)

Calcination was done after mechanic-chemical processing at 650 °C in a Thermolite 2136 air atmosphere. The programmed reduction of the temperature was conducted in order to generate oxygen vacancies in the LiNbO_3_ structure at 650 °C.

### 3.2. Powder X-ray Diffraction

The formation of the pure crystalline lithium niobate (LiNbO_3_) phase by the mechanical milling process was confirmed from XRD, using a Panalytical X-Pert system (Westborough, MA, USA) with a source of Cu_Kα_ radiation at 40 keV and 30 mA in the 2θ range between 30° and 115°, at 0.02° steps every 4 s. Crystallographic indexation was obtained using JCP: 00-074-2238 for the trigonal (R3cH). Then, using a Micromeritics 2910, a temperature-programmed reduction (TPR**)** was conducted in order to detect the structure of LiNbO_3_, under the following conditions: 5% of hydrogen and *T*_max_ of 650 °C for 20 min.

### 3.3. Magnetometry Measurements

The magnetization measurements were performed using Physical Properties Measurements (PPMS) equipment model 9T Quantum Design Vibrating Sample Magnetrometer PPMS. Both measurements were performed at 300 K.

### 3.4. Experimental HRTEM Microanalysis

Experimental imaging of ultra-high resolution TEM was performed in a JEOL ARM (200F) (Austin, TX, USA) with an operational voltage of 200 kV equipped with a Cs corrector (CEOS GmbH) (Austin, TX, USA) and an FEG-STEM/TEM unit. The high angle annular dark field (HAADF) probe size was set to 0.095 nm with a current of 23.2 pA for bright field imaging. The condenser lens aperture size was set to 40 μm. A camera length (CL) of 8 cm/6 cm and a collection angle of 68–280 mrad/90–270 mrad was set for STEM images, to eliminate contributions from un-scattered beams.

## 4. Conclusions

We present a successful solid-state reaction mechanical synthesis of LiNbO_3_ nanoparticles. The ferromagnetic effect was found for samples processed at 300 min of milling and calcination temperatures of 650 °C, as described by powder X-ray data, and with a reduction heat treatment in a 5% of hydrogen/argon atmosphere. The structural aspects show that ferromagnetic properties could exist due to voids and vacancies, since observations made in the samples subjected to heat treatment reduction show the poor stability and diffusion of single atoms, including Li and Nb, near the nanoparticle surface, as observed during STEM measurements. Thus, a possible re-orientation of the electronic structure could occur due to voids near lithium or niobium in the LiNbO_3_ crystallographic structure, as described previously using density functional methods, as presented in [12].
